# Aspirin plus verapamil relieves angina and perfusion abnormalities in patients with coronary microvascular dysfunction and Chagas disease: a pilot non-randomized study

**DOI:** 10.1590/0037-8682-0181-2021

**Published:** 2021-11-12

**Authors:** Rafael Brolio Pavão, Henrique Turin Moreira, Antonio Oswaldo Pintya, Jorge Luis Haddad, André Vannuchi Badran, Moysés de Oliveira Lima-Filho, Igor Matos Lago, João Reynaldo Abbud Chierice, André Schmidt, J. Antonio Marin-Neto

**Affiliations:** 1 Universidade de São Paulo, Faculdade de Medicina de Ribeirão Preto, Divisão de Cardiologia, Ribeirão Preto, SP, Brasil.

**Keywords:** Chagas disease, Coronary microvascular dysfunction, Cardiomyopathy, Myocardial perfusion scintigraphy, Aspirin, Verapamil

## Abstract

**INTRODUCTION:**

Most patients with chronic cardiomyopathy of Chagas disease (CCCD) harbor a secondary cause of coronary microvascular dysfunction (CMD), for which there is no evidence-based therapy. We evaluated the impact of verapamil plus aspirin on symptoms and perfusion abnormalities in patients with CCCD and CMD.

**METHODS:**

Consecutive patients with angina pectoris, who had neither coronary artery obstructions nor moderate-severe left ventricular dysfunction (left ventricular ejection fraction > 40%) despite showing wall motion abnormalities on ventriculography, were referred for invasive angiography and tested for Chagas disease. Thirty-two patients with confirmed CCCD and ischemia on stress-rest SPECT myocardial perfusion scintigraphy (MPS) were included. Clinical evaluation, quality of life (EQ-5D/ Seattle Angina Questionnaire), and MPS were assessed before and after 3 months of treatment with oral verapamil plus aspirin (n=26) or placebo (n=6).

**RESULTS:**

The mean patient age was 64 years, and 18 (56%) were female. The ischemic index summed difference score (SDS) in MPS was significantly reduced by 55.6% after aspirin+verapamil treatment. A decrease in SDS was observed in 20 (77%) participants, and in 10 participants, no more ischemia could be detected. Enhancements in quality of life were also detected. No change in symptoms or MPS was observed in the placebo group.

**CONCLUSIONS:**

This low-cost 3-month treatment for patients diagnosed with CCCD and CMD was safe and resulted in a 55.6% reduction in ischemic burden, symptomatic improvement, and better quality of life.

## INTRODUCTION

Chagas disease, caused by the protozoan *T. cruzi,* remains a public health problem not only in Latin America^1^ ^,^ ^2^, but also in non-endemic areas, such as the United States and European countries^3^ ^,^ ^4^, because of recent human migrations from endemic to developed countries. Approximately one-third of infected individuals develop chronic cardiomyopathy, the most prevalent and ominous clinical form of Chagas disease (CD)^5^ ^,^ ^6^, which is responsible for a high morbidity and mortality burden, with severe social and medical-labor impact^7^ ^,^ ^8^. Chronic cardiomyopathy of CD (CCCD) is characterized by a high frequency of anginal symptoms, progressive heart failure, thromboembolic events, malignant arrhythmia, and sudden death^9^ ^,^ ^10^. 

Approximately 20%-40% of patients with CCCD complain of angina, usually with atypical characteristics, because of its unpredictable relation to physical effort and variable duration, its occurrence often at rest, and its erratic response to nitrates. In some patients, the symptoms may be related to concomitant esophageal involvement due to CD, and sometimes it even mimics an acute coronary syndrome^11^. Although angina is a cardinal complaint in many patients with CCCD, invasive coronary angiography almost invariably shows no obstructive epicardial coronary disease^12^ ^,^ ^13^. In contrast, several structural and functional abnormalities, including left ventricular (LV) wall motion abnormalities and coronary microvascular dysfunction (CMD), which are mainly associated with inflammatory myocardial damage, have been described in both experimental models of *T. cruzi* infection and in humans with CCCD or even the indeterminate form of CD^14^ ^,^ ^15^. Based on such evidence, some patients with CCCD might have criteria for harboring a secondary type of CMD, in the context of a cardiomyopathy caused by low-grade but virtually incessant myocarditis^16^ ^-^ ^18^. Various independent investigations have shown that patients with CCCD meet the four criteria specified in recent classifications of CMD for the conundrum of patients with angina and objective signs of myocardial ischemia, normal epicardial coronary arteries on angiography, and impaired coronary microvascular function^19^ ^-^ ^23^.

The pathophysiological implications and prognostic significance of microvascular disturbances have not been entirely established in most clinical scenarios, especially in the context of primary CMD. In contrast, CMD is considered to be one of the four main mechanisms contributing to the complex pathogenesis of CCCD^24^. There is ample evidence from studies in experimental models of *T. cruzi* infection and clinical investigations using biopsy and histopathological methods, that microvascular abnormalities causing myocardial ischemia are associated with focal diffuse myocytolysis and extensive reparative fibrosis^25^. In line with several other investigations^13^ ^,^ ^14^, in a longitudinal follow-up study using myocardial perfusion scintigraphy with single photon emission computed tomography (MPS-SPECT) in patients with CCCD, the deterioration of LV systolic function over time was associated with an increase in the extent of irreversible perfusion defects^26^. Moreover, a clear topographic association was observed between the presence of ischemia in the initial evaluation and the development of myocardial fibrosis in the MPS study^26^. 

Following the recent recognition of the increasing role of CMD in the context of chronic stable coronary artery disease, despite the empirical use of therapies and sparse research initiatives^27^ ^-^ ^30^, no evidence-based therapeutic tools have been defined for patients primarily afflicted by CMD. This is even more so in patients whose CMD is attributable to CCCD.

This investigation aimed to test the hypothesis that stable patients with CCCD who had angina pectoris, normal epicardial coronary arteries on invasive angiography, and ischemic perfusion defects on MPS-SPECT could benefit from a prolonged therapeutic intervention that combined an antiplatelet agent (aspirin) and a microvascular vasodilator (verapamil).

The primary outcome of the study was set at 50% reduction of the stress-induced perfusion defect detected at baseline, to be seen after 90 days of the therapeutic intervention.

The secondary outcome was the reduction of angina and improvement in quality of life, as assessed using the Seattle Angina Questionnaire and the EQ-5D-3L (EuroQoL). 

## METHODS

We prospectively selected 272 consecutive stable patients referred to our institution for invasive coronary angiography between January 1, 2012 and December 31, 2018, whose main complaint was angina pectoris. All patients presented with normal epicardial coronary arteries but abnormal wall motion and epidemiological and clinical data suggestive of chronic CD. After this initial selection, in 68 of the patients, the diagnosis of CD was confirmed by two positive tests using distinct serological techniques. These patients were considered to have the cardiomyopathy form of CD, as they all were symptomatic, with chest pain, electrocardiogram (ECG) abnormalities, or segmental LV abnormalities on ventriculography. An additional inclusion criteria was age ≥18 years. The exclusion criteria were as follows: a) heart rate <50 bpm at rest, b) advanced AV block, c) atrial fibrillation/flutter with ventricular pre-excitation, d) systolic blood pressure >180 or <90 mmHg, and e) renal impairment (serum creatinine > 1.4 mg/dl).

**FIGURE 1:**
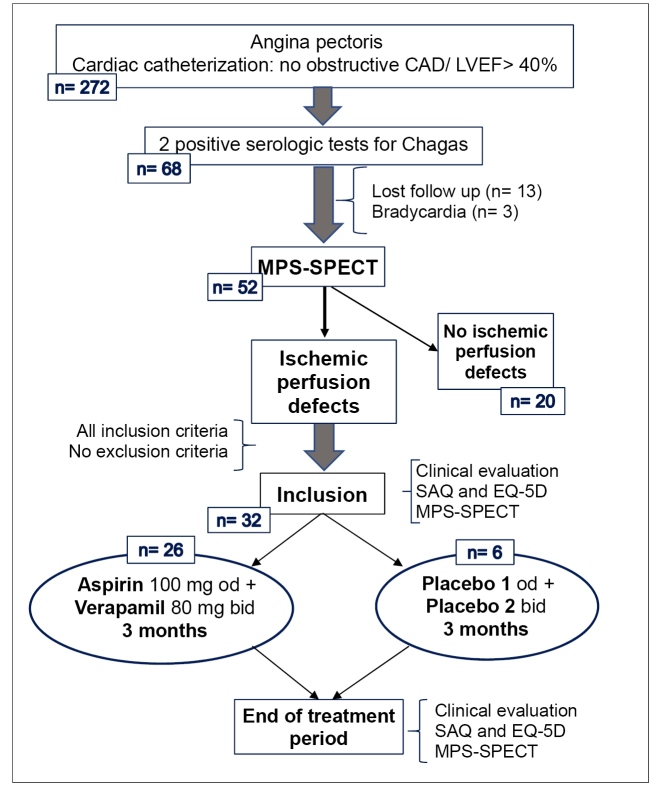
Design of therapeutic study in patients with coronary microvascular dysfunction related to chronic Chagas disease cardiomyopathy. **CAD:** coronary artery disease; **LVEF:** left ventricle ejection fraction; **MPS-SPECT:** myocardial perfusion scintigraphy, single photon emission computed tomography; **SAQ:** Seattle Angina Questionnaire.

A total of 32 patients met the following inclusion criteria: at least one ischemic perfusion defect during stress-rest MPS-SPECT to be enrolled in the therapeutic protocol of the study (Figure 1). 

Patients with a history of aspirin use prior to the study were instructed not to take the drug for 30 days before the nuclear study. None of the patients used calcium channel blockers at the time of enrollment.

These 32 patients signed the informed consent form related to the research protocol, as approved by the institutional ethics committee (No. 8430/2011). The research was included in the National Ethics of Research System (SISNEP) under the identification CAAE:181.0.000.004-11 and was carried out according to the ethical guidelines of the 1975 Declaration of Helsinki. Per protocol, all patients were also examined with a 12-lead ECG, chest radiography, transthoracic echocardiogram, and 24-h Holter monitoring to calculate the Rassi score^31^ and estimate the patient's risk of death.

## MPS-SPECT

SPECT studies were performed in all 32 patients before and after the long-term therapeutic protocol. The images were acquired in the camera range (Philips BrightView XCT - Cleveland, OH) of a double detector with the patient in the supine position during rest and stress phases. The acquisition occurred in a semicircular orbit (180°, from the right anterior oblique projection to the left posterior oblique projection), in 32 projections synchronized with the electrocardiogram, eight frames per cardiac cycle in 60 seconds by projection (“accepted” heartbeat) with a 50% acceptance window around the average R-R. The detectors were equipped with collimators of parallel holes of low energy and high resolution, using a 64 × 64 pixel acquisition matrix.

Physical exercise was used as the preferred stress test, and a vasodilator with dipyridamole had to be used in only five cases because of physical impossibility and the presence of complete left bundle branch block or pacemaker implantation. Beta-blockers, calcium channel blockers, and other anti-ischemic drugs were interrupted 48 h before the nuclear tests. Sestamibi-Tc99m was used as a radiotracer to assess regional myocardial perfusion at a dose of 12-15 mCi at rest and 25-30 mCi during stress. Images were acquired 1 hour after each intravenous injection of radiotracer.

Polar maps using the 17-segment model were generated to assess the perfusion abnormality according to a score defined as 0= normal, 1= slight uptake reduction, 2= moderate uptake reduction, 3= marked uptake reduction, and 4= no tracer uptake. Perfusion abnormalities in stress (SSS, summed stress score) and rest (SRS, summed rest score) were quantified to differentiate between reversible (summed difference score [SDS] ≥ 1) and irreversible perfusion defects (SDS=0 [SDS= SSS - SRS]). 

Scintigraphy image evaluation was always performed by two experienced researchers who were blinded to the protocol phase (before or after intervention) or treatment offered, presenting high intra- and inter-observer reproducibility (agreement= 92% for both).

### Aspirin+verapamil treatment group

Verapamil 80 mg bid and aspirin 100 mg od were prescribed to the first 26 consecutive patients for 90 days. During the treatment phase, patients were assessed for symptoms and regular use of medications at 30 days (assessment by phone) and 90 days. Symptoms of chest pain and quality of life were assessed using the Seattle Angina Questionnaire (SAQ) and EuroQol 5-Dimension 3-Level (EQ-5D-3L) during clinical evaluations on the inclusion day and after 90 days of treatment, and equally by performing MPS-SPECT (before and after the treatment protocol). 

### Placebo treatment group

Following the same inclusion criteria above, the last six consecutive subjects had no exclusion criteria and signed the informed consent form related to the research protocol, as described above. The patients in this group were blinded to the scope of this arm of the study; that is, they were not aware whether they would receive real drugs or their placebos. However, per protocol, these six patients with CMD related to CCCD were submitted to a 3-month placebo treatment. The pills taken by this group of patients had the same characteristics of verapamil and aspirin (color, taste, and visual aspect) as the drugs used in the main study. MPS, SAQ, and EQ-5D scores were assessed before and after 90 days of placebo intake, as well as a phone assessment at 30 days. 

### Statistical analysis

For the initial estimate of average SDS, we considered the results from two previous investigations in 10 similar patients with CCCD who had reversible perfusion defects: 8.1 (SD=4.98)^32^ ^,^ ^33^. We hypothesized a 50% reduction of SDS following the therapeutic intervention in paired comparison with the baseline SDS, with bicaudal alpha= 0.05 and 1-beta= 0.8. The minimum number of patients to be included was 14. With a loss of follow-up of 20%, we defined that the sample of 18 patients would be sufficient for testing our hypothesis ^34^. 

The Shapiro-Wilk tests were used to check if variables had a normal distribution, in which case paired variables were compared with Student's "t" tests; otherwise, the Wilcoxon paired test was used. Continuous variables with normal distribution were described as mean and SD, while non-normally distributed variables were described as median and IQ intervals. All tests were bicaudal at a significance level of 0.05. All analyses were performed using Stata software *(StataCorp, EUA, version 14.2)*.

## RESULTS

### Baseline characteristics

The 32 patients enrolled in the study, following the inclusion and exclusion criteria outlined above, were aged 64 (SD= 10) years, and 18 (56%) were women. All patients completed the baseline and therapeutic phases. There were no deaths, and no side effects of the medications occurred that warranted the interruption of the treatment phase (bradycardia, heart failure symptoms, or bleeding events). There was excellent adherence to the medication regimen, observed by counting empty cartons brought to medical appointments.

The risk factors for coronary artery disease and symptoms probably related to CD are described in Table 1. NYHA class I and II were found in 75% and 25% of patients, respectively, while the Rassi score was calculated as 66% of patients in the intermediate and 7% of patients in the high-risk range.

**TABLE 1:** Clinical characteristics of patients with microvascular dysfunction related to Chagas disease.

Baseline characteristics	n / %		ECG and Holter findings		n / %	
Age (years)	64 (SD=10)		Sinus rhythm		30	93,7
Female sex	18	56	Atrial fibrillation		2	2.3
HF - NYHA I	24	75	1^st^ degree AV block		5	15.6
HF - NYHA II	8	25	LBBB		3	9.4
Hypertension	23	71.8	RBBB		9	28.1
Dyslipidemia	14	43.7	Primary repolarization changes		4	12,5
Diabetes mellitus	7	21.8	Low voltage QRS complex		5	15.6
BMI> 30 Kg/m^2^	5	15.6				
Current smoking	5	15.6	Atrial tachycardia		18	56.2
Rassi score	6.8 (SD=3.2)		NSVT		6	18.7
**Symptoms related to Chagas disease**	n / %		**Echocardiogram findings**		**n / %**	
Palpitations	11	34.4	Preserved LV function		27	84.4
Intestinal constipation	8	25	Mild LV dysfunction		5	15.6
Dysphagia	4	12.5	Preserved RV function		32	100
**Medication**	n / %		**WMSI index**		1.2 IR1-1.3	
Angiotensin inhibitor	19	68.7	↑ LV mass index		9	28.1
β-blockers	14	56.2	Diastolic dysfunction		8	25
Oral hypoglycemic drugs	7	21.8	**Cardiac catheterization findings**		**n / %**	
Aspirin	16	50	LVEDP, mmHg		17.4 (SD=7)	
Statins	14	43.7	LVEDP> 15 mmHg		15	46.8
Nitrates	7	21.8	CCCD apical aneurism		10	31.2
Insulin	1	3.1	Dyssynergies	- Apex	27	84.4
			- Anterolateral		22	68.7
			-Inferolateral/ Apical inferior		18	56

**CCCD**: chronic cardiomyopathy of Chagas disease; **LVEDP:** left ventricular end-diastolic pressure; **LBBB:** left bundle branch block; **HF:** heart failure; **RBBB**: right bundle branch block; **NSTV:** non-sustained ventricular tachyarrhythmia; **WMSI**: wall motion score index; **SD**, standard deviation.

The rest of the transthoracic echocardiogram showed a mean LV ejection fraction (LVEF) of 59% (SD= 9%), with only 15% of patients having slightly depressed LVEF (between 40% and 50%) and right ventricle ejection fraction preserved in all patients. The LV mass index was elevated in 31% of patients, as defined by >95 g/m^2^ for women and >115 g/m^2^ for men. LV diastolic dysfunction was detected in 25% of the patients, while segmental systolic wall motion abnormalities appeared in 68% of the patients, with a median WMSI of 1.2 [1.0-1.3]. Echocardiography was not performed after the intervention. Cardiac catheterization findings corroborated echocardiographic data, indicating that the majority (85%) of patients presented with preserved LVEF and that all patients had at least one wall motion abnormality by contrast ventriculography, with predominance of regions usually affected by CCCD. LV diastolic dysfunction (LVEDP> 15 mmHg) was demonstrated in 46.8% of patients (Table 1).

### Myocardial perfusion scintigraphy

**FIGURE 2:**
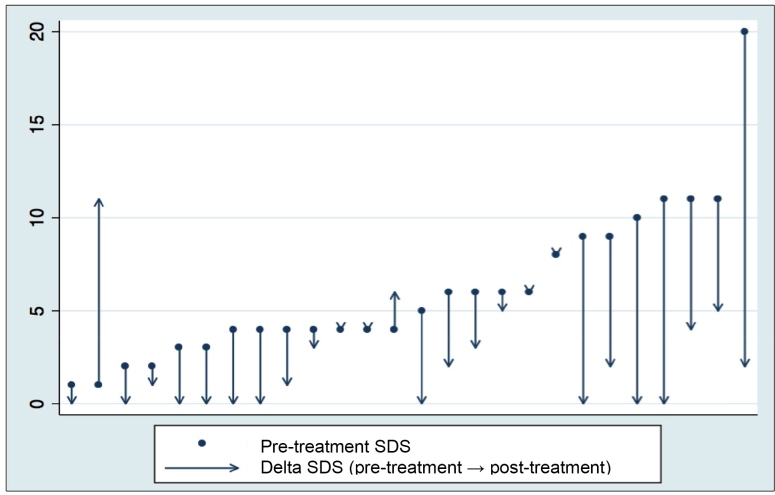
Pre- and post-treatment with aspirin plus verapamil SDS variation in individual patients studied with MPS-SPECT. **SDS**: Summed difference score; **MPS-SPECT**: Myocardial perfusion scintigraphy, single photon emission computed tomography.

In comparison to baseline evaluation, a significant 55.6% reduction in SDS was seen after aspirin+verapamil treatment, from 4.5 [4-9] to 2.0 [0-4.25], (p< 0.001). A decrease in SDS was observed in 20 participants (77%). In 10 of these patients (38.5%), a post-treatment SDS equal to zero was observed; that is, there was no evidence of stress-induced perfusion abnormalities (Figure 2).

### Analysis of symptoms and QOL analysis

The indexed values of EQ-5D increased significantly from 0.63 (SD= 0.11) at baseline to 0.77 (SD= 0.17) after aspirin+verapamil treatment (p< 0.001), as well as the visual analog scale (EQ-VAS), that was raised by 28.6% at the end of this treatment protocol (7 [6-8] vs 9 [8-10], p< 0.001). In addition, an improvement of >10 points was seen in all dimensions of QAS post-treatment (p< 0.001) as compared to baseline (Table 2).

**TABLE 2:** Results of MPS-SPECT and questionnaire scores pre- and post-treatment with aspirin and verapamil for 3 months.

MPS-SPECT scores	Pre	Post	p
*SRS* index	2 [0-4]	2.5 [0-7]	0.088
*SSS* index	6 [5-12]	5 [2-11]	0.020
*SDS* index	4.5 [4-9]	2 [0-4.25]	< 0.001
Total ischemic segments, n	158	67	N/A
Ischemic segments index	4.5 [3-9]	2 [0-4.25]	0.001
LVEF (*Gated SPECT*), %	59 [51-64]	58 [52-61]	0.135
**EQ-5D scores**	**Pre**	**Post**	**p**
Indexed EQ-5D	0.63 (SD= 0.11)	0.77 (SD= 0.17)	<0.001
EQ5-VAS	7 [6-8]	9 [8-10]	<0.001
**SAQ scores**	**Pre**	**Post**	**p**
D1- Physical limitation	77 [58-95]	95 [87-100]	<0.001
D2- Angina stability	50 [50-50]	100 [100-100]	<0.001
D3- Frequency of angina	60 [40-72.5]	80 [60-100]	<0.001
D4- Satisfaction to treatment	53 (SD= 20)	93 (SD= 8)	<0.001
D5- Perception of the disease	45 [33-66]	83 [73-92]	<0.001

SDS: summed difference score; SRS: summed rest score; SSS: summed stress score; EQ5-VAS: EuroQoL questionnaire visual analog scale; D 1-5: Dimensions 1-5.

Four patients (15.3%) maintained similar SDS values and only 2 (7.7%) had increased SDS after 3 months of medications. Rest scores (SRS) did not change significantly after the treatment protocol (Figure 3).

**Figure:**
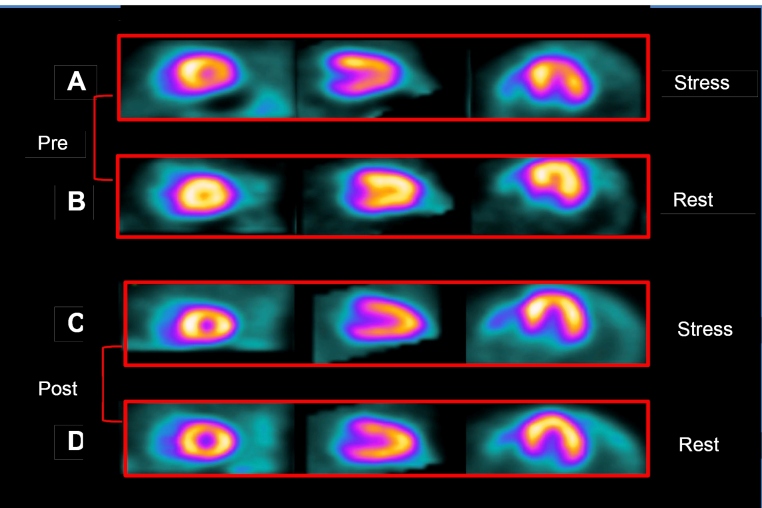

### Placebo group analysis

The baseline characteristics were similar to those in the main study. The six patients who were treated with placebo had no significant change in SDS values (5 SD= 1.9 vs 5 SD= 3.3, p= 0.24). In addition, data were obtained in QOL and angina scores, after placebo period, that were not significantly different from indexed values of EQ-5D (0.58 SD= 0.14 vs 0.6 SD= 0.14, p= 0.92) and EQ-VAS (5.5 SD= 0.83 vs 6.0 SD= 1.26, p= 0.39), as well as the five dimensions of QAS (D1: 66 SD= 25.3 vs 64.2 SD= 24.5, p= 0.57; D2: 50 SD= 0 vs 66.7 SD= 25.8, p= 0.16; D3: 70 SD= 17.9 vs 68.3 SD= 25.6, p= 0.83; D4: 47.5 SD= 10.8 vs 10.7 SD= 20.2, p= 0.09; D5: 45.8 SD= 21.6 vs 51 SD= 29.1, p= 0.4), compared to baseline.

## DISCUSSION

This is the first report to demonstrate that patients with angina pectoris and objective signs of coronary microvascular ischemia associated with CCCD can benefit from a 3-month treatment based on the combined effects of an antiplatelet and a coronary microvascular vasodilator agent. The primary benefit hypothesized in the study design was a significant decrease of >50% in myocardial perfusion ischemia induced by stress, as well as the secondary reduction of angina assessed with QAS and the improvement in the quality of life evaluated with EQ-5D. 

Based on the present evidence gathered in patients with an intermediate to high risk of death, as assessed by the Rassi score, apart from symptomatic relief, effective therapy against the development of myocardial ischemia would be beneficial in preventing the appearance of its pathophysiological consequences that have been shown to cause LV systolic deterioration in both the chronic model of animal infection with *T. cruzi* ^35^ and patients with CCCD^26^ ^,^ ^36^.

The clinical results obtained for the first time with a non-dihydropyridine calcium channel blocker in patients with coronary microvascular disease secondary to CD are in agreement with an individualized approach to manage patients with various forms of stable angina, which takes into consideration the underlying mechanism^29^. In addition, they confirmed the symptom relief that was reported with calcium channel blockers, which are considered first-line treatment for microvascular angina in patients with abnormal coronary vasodilator reserve^27^. Moreover, they corroborate previous data reported on experimental models of *T. cruzi* infection. It has been shown that treatment with a microvascular vasodilator such as verapamil slowed the progression of myocardial lesions in a murine model of CCCD^37^. Verapamil also reversed microvascular disorders (segmental spasms and capillary low flow) in the cremasteric tissue of mice infected with *T. cruzi* ^38^ ^,^ ^39^. 

Our original clinical findings regarding the use of aspirin in CMD associated with CCCD were also expected from previous experimental data on animal models. In addition to the decrease in TXA2 levels via inhibition of cyclooxygenase-1 (COX-1), low doses of aspirin in murine models infected with *T. cruzi* resulted in the reduction of endothelial adhesion molecules (ICAM and E-selectin) through COX alternative pathways^40^, leading to reduced inflammatory infiltrates and consequently myocardial damage^41^. In the hamster model of CCCD, the prolonged use of another antiplatelet vasodilator, such as dipyridamole, has been associated with a decrease in rest MPS abnormalities^15^.

Although studies correlating histopathological and myocardial scintigraphic aspects suggest the participation of microvascular abnormalities leading to fibrosis and ventricular remodeling in other dilated cardiomyopathies^42^ ^,^ ^43^, it is noteworthy that CD is a peculiar cause of secondary microvascular angina. A recent report from our institution found CCCD as a secondary cause of coronary microvascular disease in 15% of 101 stable patients whose cardinal symptoms were anginal pain warranting coronary angiography^44^. In this population sample, it was also observed that although sharing several clinical, hemodynamic, and myocardial perfusion characteristics with patients whose CMD was due to other etiologies, impairment of LV segmental and global systolic function was significantly more severe when CMD was related to CCCD. In addition to systolic dysfunction, most patients with CMD related to CD exhibited LV diastolic dysfunction detected invasively by high LVEDP at rest^20^. These data indicate that such patients may be at risk of developing heart failure with preserved ejection fraction, as recently described in patients with impaired coronary flow reserve and LV diastolic dysfunction^44^. In addition to Chagas cardiomyopathy, some patients enrolled in this study presented with other risk factors for obstructive coronary artery disease and CMD^45^ ^,^ ^46^ such as hypertension, dyslipidemia, diabetes mellitus, and LV hypertrophy. As confounding factors for evaluating the effects of CMD associated with CCDC treatment, stratified analysis for each of these comorbidities was performed and no significant changes were verified in the final results. They also exhibited marked elevation of LV end-diastolic pressure as a sign of LV diastolic dysfunction. Although by protocol moderate/severe global LV systolic dysfunction was excluded, nearly 70% of patients enrolled had LV segmental abnormalities on echocardiogram, a hallmark of CCCD. Therefore, the promising results of our trial lend support to the hope that microvascular derangements, which are associated with myocardial ischemia that potentiates and amplifies the chronic inflammatory process^36^, eventually leading to coalescent microinfarctions and the development of typical aneurysms often seen in patients with CCCD, can be averted with the treatment regimen proposed here^24^. 

In the context of CD, abnormal microvascular reactivity has been reported in vascular territories other than the coronary bed, to a similar extent to that found in patients with obstructive coronary artery disease^21^. Derangement of coronary microvascular endothelial function is also suggested to occur early in the natural history of CD, as shown by a study using stress echocardiography that found a reduced coronary flow reserve to the vasodilator dipyridamole in Chagas patients with the indeterminate form, that is, even before the cardiomyopathy is clinically apparent^22^. These findings are corroborated by the detection of rest and stress-induced myocardial perfusion abnormalities, at least in some patients with the indeterminate form of CD^47^ ^,^ ^23^. Furthermore, they are in agreement with the possibility that early impairment of myocardial perfusion caused by microvascular disturbance eventually leads to myocardial fibrosis, as shown by recent studies using magnetic resonance in patients with the indeterminate form of CD^48^.

Our results highlight the relevance of defining different pathogenic mechanisms and diagnoses in the genesis of CMD, such that specific therapeutic options can be devised accordingly^49^ ^,^ ^50^. Such mechanisms entail the application of appropriate diagnostic tools to characterize not only secondary causes of CMD, but also define the extent of myocardial damage in the context of diverse diseases that could be amenable to adequate therapeutic measures.

At the time of the conception of this clinical trial in 2011, the criteria for CMD were not well established^51^. The international standardization came in 2017, including four criteria: (1) presence of symptoms suggestive of myocardial ischemia, (2) objective documentation of myocardial ischemia, (3) absence of obstructive CAD (coronary diameter reduction <50% and/or fractional flow reserve - FFR ≤0.80), and (4) confirmation of a reduced coronary blood flow reserve and/or inducible microvascular spasm^50^. Concerning this fourth requirement, although reduced coronary flow reserve has been previously shown in patients with CD^44^, in our study no direct assessment of coronary microvascular function was performed for the patients with suspected microvascular angina. 

Our study did not include a placebo phase for all the enrolled patients. However, the findings in the subset of six individuals who received the placebo for the same period as the main subset of 26 patients effectively treated showed not only the absence of symptomatic response, but also the persistence of myocardial perfusion defects. The study was only registered on the Brazilian platform and not registered in any international registry system for clinical trials before including patients.

Finally, even though the benefit of etiology therapy has not been definitively proven after the cardiomyopathy is established^52^ ^,^ ^53^, the lack of specific treatment in all patients recruited into this investigation may raise some concern, since recent research indicates that the progression of CD may be deterred when individuals with the early stages of cardiomyopathy or the indeterminate form are given benznidazole^54^. 

This clinical trial points to the possibility of a new beneficial low-cost treatment for patients diagnosed with CD and CMD that seems to be safe, with no adverse effects reported 3 months after the combined use of aspirin and verapamil, resulting in a 55.6% reduction in ischemic burden by myocardial scintigraphy and improvement in symptoms and quality of life. Further work will be necessary to reinforce the applicability of this treatment and compare its results with those from a larger placebo group.
